# Choroidal vascular changes in internal carotid artery stenosis: a retrospective cohort study in Chinese population

**DOI:** 10.1186/s12886-019-1218-7

**Published:** 2019-11-07

**Authors:** Shuang Li, Xuqiang Lang, Wei Wang, Yang Yang, Jingjing Wang, Hongyang Li, Yanling Wang, Kang Wang

**Affiliations:** Department of Ophthalmology, Capital Medical University, Affiliated Beijing Friendship Hospital, 95 Yongan Road, Xicheng District, Beijing, 100050 China

**Keywords:** Internal carotid artery, Spectral domain optical coherence tomography, Binarization tool, Choroidal vascularity index

## Abstract

**Background:**

To evaluate choroidal vascularity index (CVI) in eyes with internal carotid artery (ICA) stenosis using binarization tool in enhanced depth images scanned by spectral domain optical coherence tomography.

**Methods:**

A retrospective cohort study was conducted in 142 patients with ICA stenosis, and 20 normal control subjects matched with the age, sex, etc. According to the diagnostic criteria, the participants are divided into a normal control group (20 cases), a mild stenosis group (64 cases), a moderate stenosis group (61 cases), and a severe stenosis group (17 cases). Enhanced depth imaging optical coherence tomography (EDIOCT) was performed to scan macular fovea, which was separated into a luminal area and a stromal area using image binarization. The choroidal vascularity index (CVI) is luminal area divided by total choroidal area.

**Results:**

There was no statistical difference in age or sex among groups. Subfoveal choroidal thickness (SFCT) in the severe stenosis group was significantly lower than that in the normal group (*P* < 0.05). Moreover, the CVI in moderate stenosis group and severe stenosis group were significantly lower compared with the normal control group (*P* < 0.001). When CVI = 65.16% was set as the cut-off value, all 162 subjects could be divided into two groups, CVI ≤ 65.16 (*n* = 83) and CVI > 65.16% (*n* = 79). The proportions of mild stenosis, moderate stenosis, and severe stenosis in CVI ≤ 65 .16 group and CVI > 65.16% group were statistically significant (*P* < 0.001).

**Conclusion:**

CVI may be a useful indicator for early diagnosis and monitoring of choroidal vascular changes in ICA stenosis.

## Background

Internal carotid artery (ICA) stenosis or obstruction may lead to severe ischemic cerebrovascular disease, and is also an important cause of ischemic eye disease, which not only affects the patient’s vision, but even causes physical disability and sudden death. When ICA stenosis is greater than 50%, the incidence of ocular symptoms increases [1, 2]. Hemodynamic changes in patients with carotid stenosis include retinal vascular occlusion, normal tension glaucoma, and peripheral retinal hemorrhage, which were about 1.8-fold, 1.9-fold, and 2.4-fold higher than normal controls, respectively. About 5% of patients with severe ICA stenosis develop ocular hemodynamic abnormalities that can lead to neovascular glaucoma, optic atrophy, and even permanent blindness. As reported, 7.5 out of every 1 million people develop ocular ischemic syndrome [3]. However, this proportion may be underestimated because the correlation between ocular manifestations and carotid stenosis is often not noticed by doctors and patients [4, 5]. Previous studies have focused more on ocular changes and retinal vascular changes in ICA patients. However, the choroid is the most important part sustaining for the ocular blood flow. When ICA occurs, choroidal blood flow is bound to be affected. Therefore, choroidal blood flow is likely to be the first sensitive indicator of blood flow change [6].

With the emergence of new optical coherence tomography (OCT) techniques, the study of choroidal structure has made great improvement [3, 6, 7]. Swept-source optical coherence tomography uses longer wavelengths and faster scanning speeds for deep imaging and quantitative assessment of the choroid. Choroidal thickness was used thereafter to represent choroidal blood flow [8]. Choroidal thickness may be affected by physiological and pathological changes [9–12]. Moreover, choroidal thickness does not provide us with information regarding the changes of choroidal structure. The change in choroidal thickness may be due to vascular system, as well as stromal tissue. Therefore, more stable and accurate diagnostic indicators are required to assess choroidal vascular status.

The application of choroidal structure image binarization to investigate the structure of the choroid and its changes in different disease models have been investigated in recent years. A number of studies have demonstrated that choroidal structure image binarization are rarely affected by physiological variables and are capable of providing choroidal vascular information [13–19]. In our study, we used the choroidal vascular index (CVI) to represent the choroidal vascular changes, and to determine differences in the proportion of choroidal vasculature in patients with ICA stenosis and healthy controls.

## Methods

### Population

This retrospective cohort study was conducted in the Department of Ophthalmology, Beijing Friendship Hospital from January 2016 to December 2017. A total of 179 patients diagnosed with ICA stenosis/obstruction were enrolled and divided into four groups according to the North American Symptomatic Carotid Endarterectomy Trial (NASCET) criteria [20]: a non-stenosis group (normal control), a mild stenosis group, a moderate stenosis group, and a severe stenosis group. Fundus photography and spectral-domain optical coherence tomography (SD-OCT) were applied in each patient with the carotid stenosis.

NASCET stenosis classification method was used to calculate the degree of stenosis according to the following formula: stenosis degree% = (1-diameter of the narrowest vessel lumen/diameter of the normal lumen at the distal end of the stenosis)*100%. The diameter (N) of the narrow part and the diameter (D) of the distal normal vessels were measured. The stenosis rate r = (1-N/D) × 100%. Stenosis was graded: no stenosis (stenosis rate 0), mild stenosis (stenosis rate < 29%), moderate stenosis (stenosis rate 30–69%), severe stenosis (stenosis rate 70–99%) and complete occlusion.

### Inclusion criteria and exclusion criteria

ICA stenosis or plaque formation was confirmed by head- and neck- CT. The ICA patients with following ocular characteristics were excluded: diopter > ± 3.0 (may affect visual acuity), active intraocular inflammation and/or infection, or any type of intraocular surgery (except cataract surgery) and retinal neovascularization caused by diabetic retinopathy or other macular disease. Additionally, the patients with the systemic diseases were also excluded: patients with peripheral vascular disease, limb paralysis, severe eye disease history (corneal disease, glaucoma, macular degeneration, ocular trauma, pathological myopia and severe cataract); acute myocardial infarction, stroke and infectious inflammatory disease in the first few weeks of the study; patients with persistent heart rate abnormalities and chronic heart failure; pulmonary hypertension and patients with acute or chronic infectious diseases or malignant tumors; obvious refractive interstitial opacity or abnormality affecting OCT measurement, such as obvious cataract, vitreous opacity, vitreous hemorrhage, vitreous cavity silicone oil filling state, etc.; patients who have undergone internal carotid artery surgery.

### Data collection

A total of 159 patients and 20 control subjects were included in the data collection. The data included the following contents: general information (age, occupation, marital status, education level, work intensity); health status (gender, hypertension, diabetes, coronary atherosclerotic heart disease, hyperlipidemia, cerebrovascular disease, malignant tumor, peripheral vascular disease, etc.); daily life habits (drinking, sleeping, smoking, drinking tea, physical exercise); eating habits (halophilic, fruit, vegetables and meat); and family history (with hypertension, diabetes and coronary heart disease).

Ocular data were collected from all enrolled patients, including best corrected visual acuity (BCVA), non-contact intraocular pressure measurement, slit-lamp ophthalmoscopy, color fundus photography, enhanced depth imaging optical coherence tomography (EDIOCT) (Heidelberg Spectalis OCT, scan mode 10 mm). The OCT image acquisition time was 9–12 AM daily.

### Quality control of image

The artery occlusion was selected on the heavier side. One eye with clear choroidal image was selected for analysis in normal subjects and the patients with the similar occlusion in both sides. All data were collected by the same technician (Wang Wei). After the EDIOCT, the best image was displayed on a computer screen and independently evaluated by three physicians. When two or more graders determine that the foveal choroid image was clearly identifiable, the image was considered acceptable and used for analysis. For pictures with unclear choroidal image, two physicians explored the boundary. If there were significant discrepancies between the two, the third physician was inquired. Finally, 17 images were excluded, and the remaining 162 images were obtained from the four groups, including no carotid artery stenosis group (20 cases), mild stenosis group (64 cases), moderate stenosis group (61 cases), and severe stenosis group (17 cases).

### Range of subfoveal choroidal area

**Fig. 1:**
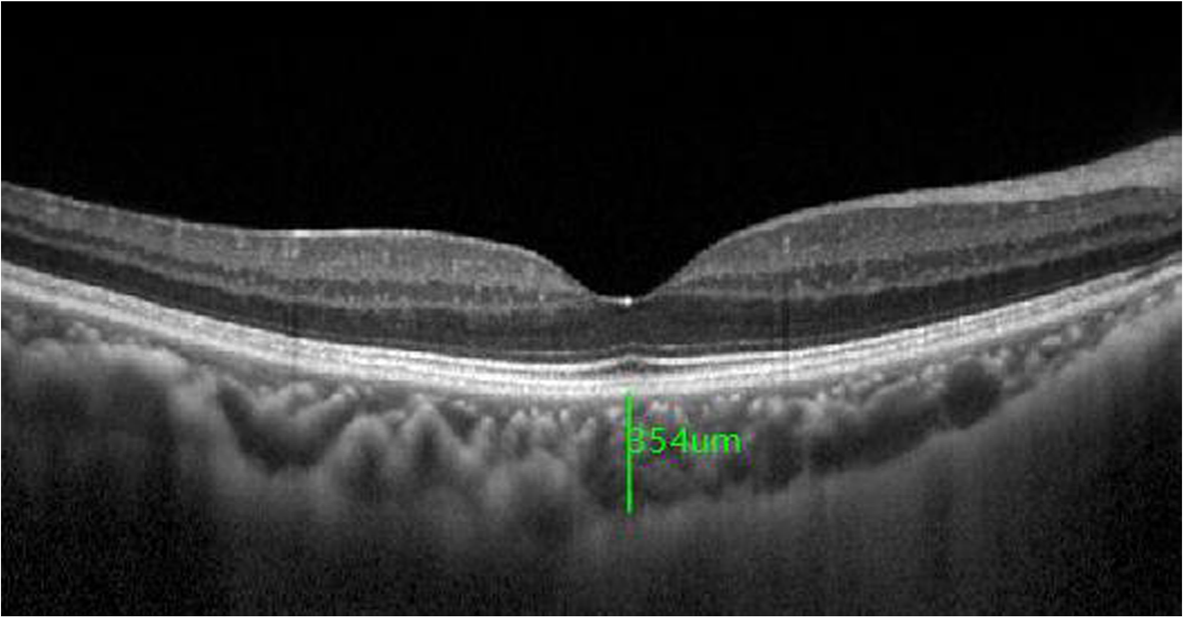
Subfoveal choroidal thickness (SFCT) was measured in the fovea of ​​the macula. It was the distance from the outer surface of the retinal pigment epithelial layer to the choroid and sclera interface below the fovea

Subfoveal choroidal thickness (SFCT) was measured in the fovea of the macula. It was the distance from the outer surface of the retinal pigment epithelial layer to the choroid and sclera interface below the fovea (Fig. 1). The nasal side is 750 μm with the fovea as the starting point, and the temporal border is 750 μm with the fovea as the starting point.

### Image binarization

**Fig. 2:**
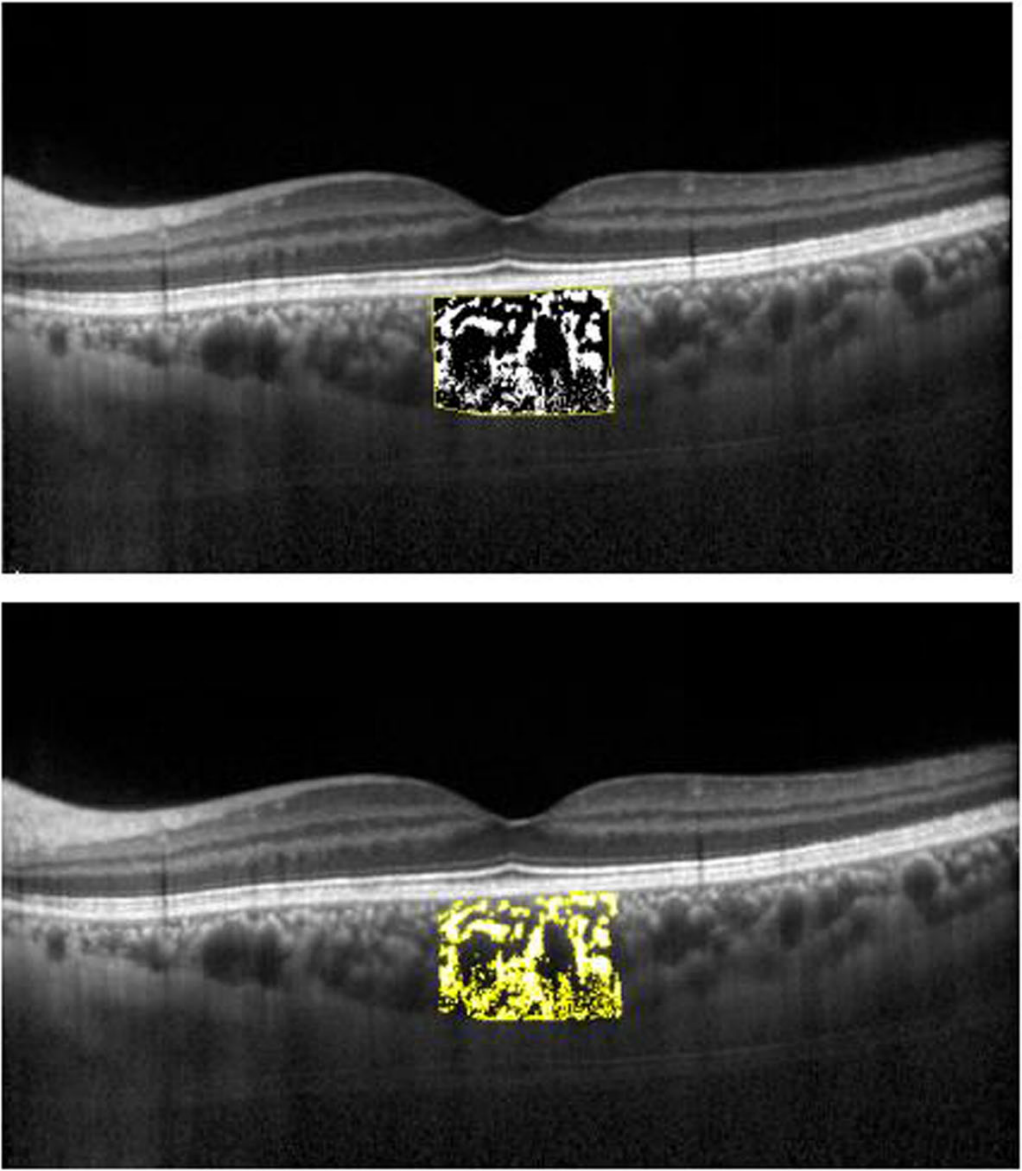
Enhanced depth images obtained using spectral domain OCT of a patient. A) EDIOCT scanning of a patient with ICA. B) Overlay image of EDIOCT scan with region of interest obtained after image binarization

Using the Auto Local Threshold (Niblack Method) and polygon selection tool, the choroidal region was marked with bruch membrane as the upper boundary and choroidal-scleral interface as the lower boundary, with the central fovea as the center, and the distances between nasal and temporal sides were 750 μm. The total choroidal area (TCA) was calculated, and the image was converted into 8-bit. The Auto Local Threshold was used to perform image binarization. In the binarized image, dark pixels represented the vascular cavity, and white pixels represented stromal area. After converting the image into RGB (red, green, blue) color, the dark pixels were selected using the color threshold tool to calculate the area of ​​the dark pixels of the luminal area (LA). Stromal area (SA) was calculated by subtracting LA from the TCA. The ratio of LA to TCA is defined as CVI (Fig. 2).

### Data analysis

The data was analyzed and processed using SPSS 19.0 software. Countable data were analyzed by χ2 test. One-way ANOVA was used to compare the data among groups, and the data were expressed as mean ± standard deviation (SD). The difference was statistically significant when *P* values were less than 0.05.

## Results

A total of 162 eyes were included in the study, 20 eyes in the normal control group, and 142 eyes in the ICA stenosis groups, including 64 cases in mild stenosis group, 61 cases in moderate stenosis group, and 17 cases in severe stenosis group. In the control group, there were 12 males (60%) and 8 females (40%); 43 males (67.2%) and 21 females (32.8%) in the mild carotid stenosis group; there were 42 males (68.9%) and 17 females (30.4%) in the moderate stenosis group. There were 10 males (58.8%) and 7 females (41.2%) in the severe stenosis group. The gender distribution of the four groups was not statistically significant (*P* = 0.734). There was no significant difference in the mean age among the four groups (*P* = 0.892). There were no differences regarding the incidence rates of diabetes, cerebrovascular disease coronary heart disease in four groups. Therefore, we ignored the impact of these factors on the final analysis (Table 1).

**Table 1:** Characteristics of participants

Groups	Normal (*n* = 20)	Mild stenosis (*n* = 64)	Moderate stenosis (*n* = 61)	Severe stenosis (*n* = 17)	*P*
Gender, male (%)	12 (60%)	43 (67.2%)	42 (68.9%)	10 (58.8%)	0.734
Age, yrs	65.15 ± 6.14	64.22 ± 6.61	64.43 ± 6.68	6.682 ± 5.27	0.892
Hypertension (%)	8 (40%)	20 (31.3%)	14 (23%)	8 (47.1%)	0.198
Diabetes (%)	4 (20%)	10 (15.6%)	11 (18%)	4 (23.5%)	0.884
Cerebrovascular disease (%)	2 (10%)	6 (9.3%)	10 (16.4%)	5 (29.4%)	0.173
CHD (%)	2 (10%)	6 (9.3%)	10 (16.4%)	4 (23.5%)	0.387
Hyperlipidaemia (%)	5 (25%)	9 (14.1%)	10 (16.4%)	6 (35.3%)	0.191
Current smoker (%)	5 (25%)	10 (15.6%)	9 (14.8%)	4 (23.5%)	0.638
Alcohol consumption (%)	5 (25%)	11 (17.2%)	11 (18%)	6 (35.3%)	0.361

*CHD* Coronary artery heart disease

SFCT was analyzed in the control group and carotid stenosis group. The SFCT values in normal control group, mild stenosis group, moderate stenosis group and severe stenosis group was 251.86 ± 35.2 μm (95% CI: 235.38–268.34), 259.25 ± 30.07 μm (95% CI: 253.73–266.76), 252.42 ± 30.78 μm (95% CI: 244.53–260.30), and 230.32 ± 30.80 μm (95% CI: 214.4–246.16), respectively. There was no significant difference between the ICA mild stenosis group and the ICA moderate stenosis group compared with the normal control group (*p* = 0.355 and *p* = 0.945). The significant difference was only observed between control and severe stenosis group (*P* < 0.05) (Table 2).

**Table 2:** Choroidal and retinal parameters between healthy controls and stenosis group

Groups	Normal (*n* = 20)	Mild stenosis (*n* = 64)	Moderate stenosis (*n* = 61)	Severe stenosis (*n* = 17)	*P*
SFCT, μm	251.86 ± 35.20	259.25 ± 30.07	252.42 ± 30.78	230.32 ± 30.80	0.01
TCA, mm^2^	1.06 ± 0.14	1.08 ± 0.12	1.07 ± 0.11	0.94 ± 0.11	0.001
SA, mm^2^	0.35 ± 0.06	0.36 ± 0.05	0.38 ± 0.04	0.34 ± 0.07	0.013
LA, mm^2^	0.71 ± 0.09	0.72 ± 0.080	0.69 ± 0.07	0.59 ± 0.06	0.001
CVI (LA/TCA), %	66.84 ± 1.45	66.39 ± 1.8	64.42 ± 0.9	62.76 ± 1.01	0.001

**Fig. 3:**
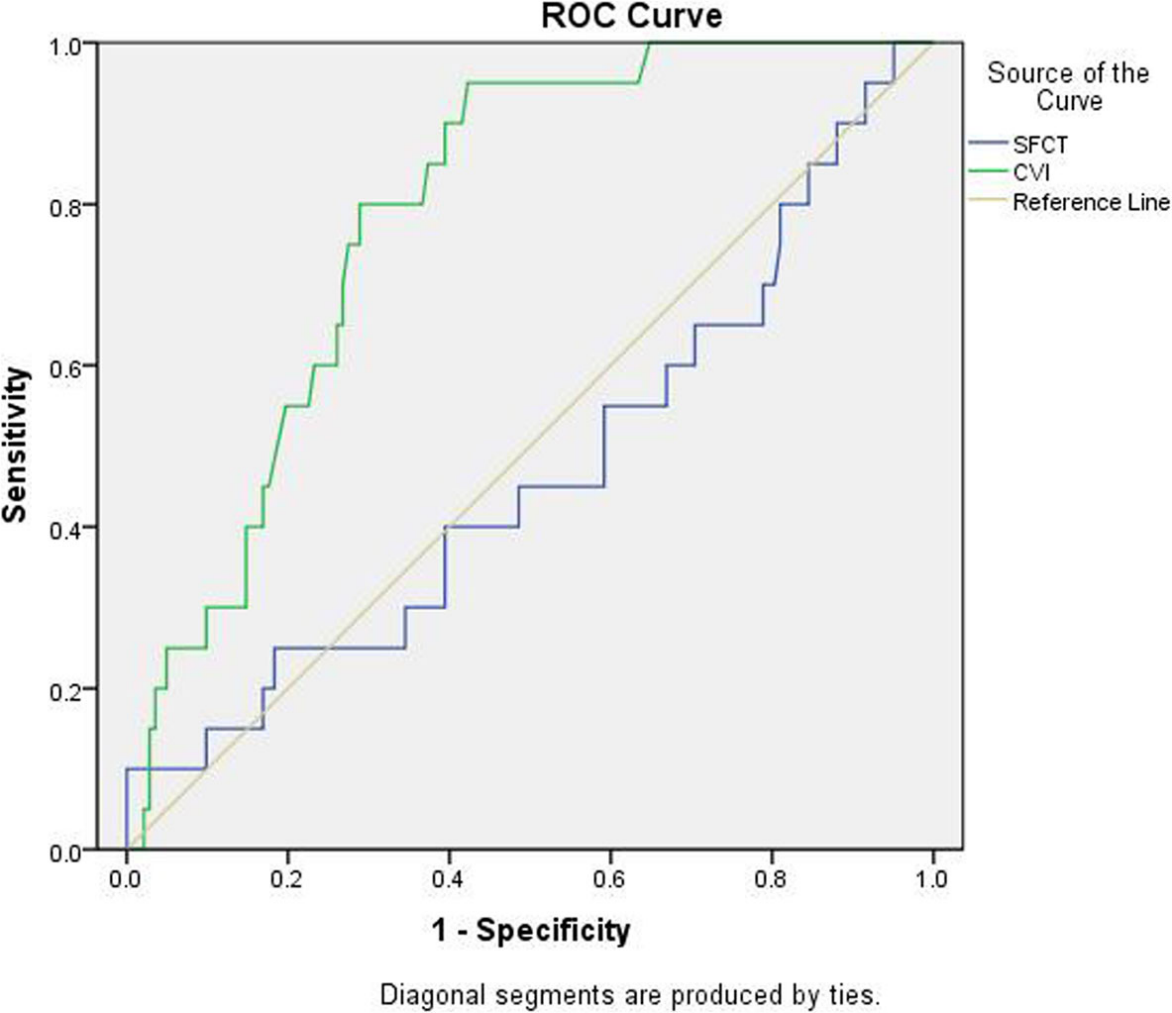
The receiver operating characteristic (ROC) curve. The area under the ROC curve of CVI was 0.788. The specificity and sensitivity of CVI were higher than those of SFCT. The Youden index was 65.16%

The CVI of the normal control group was 66.84 ± 1.45% (95% CI: 66.16–67.51%), 66.39 ± 1.8% (95% CI: 65.94–66.83%) in the ICA mild stenosis group, 64.42 ± 0.9% (95% CI: 64.19–64.65%) in the ICA moderate stenosis group and 62.76 ± 1.01% (95% CI: 62.24–63.28%) in the ICA severe stenosis group (Table 2). The CVI of the ICA mild stenosis group was lower than that of the normal control group (Table 2), but the difference was not statistically significant (*P* = 0.219). When compared with normal control group, the values in ICA moderate stenosis group and ICA severe stenosis group were statistically reduced (*P* < 0.001). The receiver operating characteristic (ROC) curve was shown in Fig. 3. The area under the ROC curve of CVI was 0.788. The specificity and sensitivity of CVI were higher than those of SFCT. The Youden index was 65.16%. Thus, this value was considered as the critical value of CVI in carotid stenosis group and normal control group (Fig. 3).

We used CVI = 65.16% as the cut-off value. All 162 participants were divided into two groups, CVI ≤ 65.16% (*n* = 83) and CVI > 65.16% (*n* = 79) (Table 3). In the CVI ≤ 65.16% group, there were 53 males (63.9%). In the CVI > 65.16%, there were 55 males (69.6%) (*P* = 0.912). There was no significant difference in the mean age between the two groups (*P* = 0.604). There was also no significant difference in the proportion of hypertension, diabetes, cerebrovascular disease and coronary heart disease between the two groups. Therefore, we ignored the impact of these factors on the final analysis.

**Table 3:** Characteristics of participants

Groups	CVI ≤ 65.16% (*n* = 83)	CVI > 65.16% (*n* = 79)	*P*
Gender, male (%)	53 (63.9%)	55 (69.6%)	0.912
Age, yrs	64.34 ± 6.38	64.87 ± 6.5	0.604
Hypertension (%)	25 (30.1%)	25 (31.6%)	0.834
Diabetes (%)	14 (16.9%)	15 (19%)	0.954
Cerebrovascular disease (%)	10 (12%)	13 (16.5%)	0.584
CHD (%)	10 (12%)	11 (13.9%)	0.738
Hyperlipidaemia (%)	12 (14.5%)	18 (22.8%)	0.287
Current smoker (%)	13 (15.7%)	15 (19%)	0.786
Alcohol consumption (%)	16 (19.3%)	17 (21.5%)	0.971

*CHD* Coronary artery heart disease

CVI ≤ 65.16% group included 1 normal subject, 18 ICA mild stenosis, 47 moderate ICA stenosis and 17 patients with ICA severe stenosis. CVI > 65.16% group included 19 normal subject, 46 patients with ICA mild stenosis, 14 patients with ICA moderate stenosis, 0 patients with ICA severe stenosis. The distribution of the patients in two groups had statistical significance (*P* < 0.001) (Table 4).

**Table 4:** Choroidal and retinal parameters between healthy controls and stenosis group

Groups	CVI ≤ 65.16% (*n* = 83)	CVI > 65.16% (*n* = 79)	*P*
Normal group	1 (1.2%)	19 (24.1%)	< 0.001
Mild stenosis group	18 (21.7%)	46 (58.2%)	
Moderate stenosis group	47 (56.6%)	14 (17.7%)	
Severe stenosis group	17 (20.5%)	0	

## Discussion

In our study, EDIOCT was applied to scan macular fovea and SFCT was measured. Image binarization tool was utilized to calculate CVI in patients with ICA stenosis. Interestingly, our data implicated CVI as a more accurate parameter to diagnose or monitor choroidal vascular changes in ICA stenosis.

ICA stenosis can cause changes in ocular hemodynamics [21]. The choroid blood flow is the most abundant part of the blood flow in the eye. When ICA stenosis occurs, the flow of choroidal blood is affected, which may be the first sensitive indicator of changes of blooding flow [22]. Due to the limitations of examination methods, there are few studies on the choroid, and the qualitative and quantitative observation of the choroid is extremely difficult using spectral domain optical coherence tomography or even swept-source OCT [23]. Histologically, the choroid is composed of blood vessels and stroma (pigment cells, smooth muscles, neurons, blood vessel walls and connective tissue). Therefore, we should not only observe changes in choroidal thickness. Instead, vascular structure may be more sensitive indicator. To determine this, it is necessary to conduct morphometric analysis. However, since the choroid is basically composed of blood vessels and has no uniform or fine structure like the retina, it is quite difficult to perform morphometric analysis.

Branchini et al. [3] firstly described how to analyze OCT images to determine the choroidal vasculature and use custom software to calculate the ratio of bright to dark pixels. Sonoda et al. [13, 14] used binarization techniques to distinguish between the choroidal vascular luminal area and the choroidal stromal area, further confirming that dark regions represent vascular structures, while bright regions represent stromal portions. Agrawal et al. [15, 17–19] used the concept of CVI to evaluate the vascular state of the choroid. Compared with SFCT, CVI was less affected by other factors. SFCT was the only factor associated with CVI, and the choroidal stromal area was not significantly associated with SFCT. In studies of various diseases, measuring the proportion of choroidal vessels provided more effective information for clinical work.

In our study, SFCT was significantly lower in the ICA severe stenosis group, which is consistent with our previous findings (5), while the SFCT in the mild stenosis group and the moderate stenosis group was comparable with the normal group. The CVI of the severe stenosis group was significantly lower than that of the normal group, indicating that the choroidal thickness did not change significantly during moderate stenosis, but the ratio of vascular components in the choroid had changed greatly. The area of LA was larger than that of the normal group. However, the area of SA was significantly lower than that of the normal group. The blood supply to the eye tissue mainly comes from the ophthalmic artery. The reduction of blood supply to the ophthalmic artery will inevitably affect the retina and choroid. Choroidal blood flow accounts for 90% of ocular blood flow, and has important physiological effects.

We hope to find a diagnostic indicator to detect changes in ocular blood flow in ICA patients. A CVI of 65.16% was set as a cut-off value after analyzing the ROC curve and all cases were divided into two groups. The composition between the two groups was significantly different. CVI > 65.16% group mainly included normal control group, ICA mild stenosis cases and a small portion of moderate stenosis cases, but without severe stenosis cases. CVI ≤ 65.16% group mainly included patients with moderate stenosis group and all patients with severe stenosis group. A small number of patients with mild stenosis were included in CVI ≤ 65.16% group, which also confirmed our hypothesis that when the ICA stenosis is aggravated, the eye presents a chronic ischemic state, which not only affects the choroidal capillaries, but also reduces the diameter. It affects the small blood vessels in the Haller’s layer, and the inner layer of the Sattler layer, resulting in a decrease in the proportion of the choroidal vasculature.

Our study has several limitations. Firstly, all the observers enrolled in this study were from hospital patients, and the prevalence of various cardiovascular diseases such as cardiovascular and cerebrovascular diseases was slightly higher than that of the normal population, although without significant difference when compared with controls. In addition, bias might be caused by the small number of patients in the severe stenosis group. The future study will expand the enrollment group, especially those in the severe stenosis group. Secondly, we only measured a single scan of the 1500-μm region centered on the fovea, and a larger region or macular volume scan may provide us with better choroidal vascular index information.

## Conclusion

In summary, the use of choroidal image binarization to analyze choroidal structural changes in patients with ICA stenosis provides us with a possibility to correlate the choroidal vasculature with the large vascular system of the body. The patients with CVI ≤ 65% have a greater possibility of serious occurrence.

## Data Availability

Not applicable.

## References

[1] Mizener JB, Podhajsky P, Hayreh SS (1997). Ocular ischemic syndrome. Ophthalmology..

[2] Liu Z, Zhao L, Xie G, Wang J, Wang Y, Feng Y (2014). Evaluating the risk of ischemic cerebral vascular disease with ocular hemodynamics in carotid artery stenosis patients. Chin J Ophthal.

[3] Branchini LA, Adhi M, Regatieri CV, Nandakumar N, Liu JJ, Laver N (2013). Analysis of choroidal morphologic features and vasculature in healthy eyes using spectral-domain optical coherence tomography. Ophthalmology..

[4] Yatsuya H, Folsom AR, Wong TY, Klein R, Klein BE, Sharrett AR (2010). Retinal microvascular abnormalities and risk of lacunar stroke: atherosclerosis risk in communities study. Stroke..

[5] Wang H, Li H, Zhang X, Qiu L, Wang Z, Wang Y (2017). Ocular image and Haemodynamic features associated with different Gradings of Ipsilateral internal carotid artery stenosis. J Ophthalmol.

[6] Wakatsuki Y, Shinojima A, Kawamura A, Yuzawa M (2015). Correlation of aging and segmental Choroidal thickness measurement using swept source optical coherence tomography in healthy eyes. PLoS One.

[7] Cole ED, Novais EA, Louzada RN, Moult EM, Lee BK, Witkin AJ (2016). Visualization of changes in the Choriocapillaris, Choroidal vessels, and retinal morphology after focal laser photocoagulation using OCT angiography. Invest Ophthalmol Vis Sci.

[8] Okamoto M, Matsuura T, Ogata N (2015). Choroidal thickness and choroidal blood flow after intravitreal bevacizumab injection in eyes with central serous chorioretinopathy. Ophthalmic Surg Lasers Imaging Retina.

[9] Ferrara D, Waheed NK, Duker JS (2016). Investigating the choriocapillaris and choroidal vasculature with new optical coherence tomography technologies. Prog Retin Eye Res.

[10] Vupparaboina KK, Nizampatnam S, Chhablani J, Richhariya A, Jana S (2015). Automated estimation of choroidal thickness distribution and volume based on OCT images of posterior visual section. Comput Med Imaging Graph.

[11] Adhi M, Brewer E, Waheed NK, Duker JS (2013). Analysis of morphological features and vascular layers of choroid in diabetic retinopathy using spectral-domain optical coherence tomography. JAMA Ophthalmol.

[12] Ruiz-Medrano J, Flores-Moreno I, Pena-Garcia P, Montero JA, Duker JS, Ruiz-Moreno JM (2015). Asymmetry in macular Choroidal thickness profile between both eyes in a healthy population measured by swept-source optical coherence tomography. Retina..

[13] Sonoda S, Sakamoto T, Yamashita T, Uchino E, Kawano H, Yoshihara N (2015). Luminal and stromal areas of choroid determined by binarization method of optical coherence tomographic images. Am J Ophthalmol.

[14] Sonoda S, Sakamoto T, Yamashita T, Shirasawa M, Uchino E, Terasaki H (2014). Choroidal structure in normal eyes and after photodynamic therapy determined by binarization of optical coherence tomographic images. Invest Ophthalmol Vis Sci.

[15] Agrawal R, Gupta P, Tan KA, Cheung CM, Wong TY, Cheng CY (2016). Choroidal vascularity index as a measure of vascular status of the choroid: measurements in healthy eyes from a population-based study. Sci Rep.

[16] Agrawal R, Chhablani J, Tan KA, Shah S, Sarvaiya C, Banker A (2016). Choroidal vascularity index in central serous Chorioretinopathy. Retina..

[17] Agrawal R, Li LK, Nakhate V, Khandelwal N, Mahendradas P (2016). Choroidal vascularity index in Vogt-Koyanagi-Harada disease: an EDI-OCT derived tool for monitoring disease progression. Transl Vis Sci Technol.

[18] Agrawal R, Salman M, Tan KA, Karampelas M, Sim DA, Keane PA (2016). Choroidal vascularity index (CVI)--a novel optical coherence tomography parameter for monitoring patients with Panuveitis?. PLoS One.

[19] Agrawal R, Xin W, Keane PA, Chhablani J, Agarwal A (2016). Optical coherence tomography angiography: a non-invasive tool to image end-arterial system. Expert Rev Med Devices.

[20] HJM B, Taylor DW, Haynes RB, Sackett DL, Peerless SJ, North American Symptomatic Carotid Endarterectomy Trial C (1991). Beneficial effect of carotid endarterectomy in symptomatic patients with high-grade carotid stenosis. N Engl J Med.

[21] Ma F, Su J, Shang Q, Ma J, Zhang T, Wang X (2018). Changes in ocular hemodynamics after carotid artery angioplasty and stenting (CAAS) in patients with different severity of ocular ischemic syndrome. Curr Eye Res.

[22] Barkana Y, Harris A, Hefez L, Zaritski M, Chen D, Avni I (2003). Unrecordable pulsatile ocular blood flow may signify severe stenosis of the ipsilateral internal carotid artery. Br J Ophthalmol.

[23] Asensio-Sanchez VM (2016). SD-OCT findings in polypoidal choroidal vasculopathy. Arch Soc Esp Oftalmol.

